# Seroprevalence of hepatitis A virus antibodies among children and adolescents living in Northern Thailand: an implication for hepatitis A immunization

**DOI:** 10.1038/s41598-023-44643-0

**Published:** 2023-10-13

**Authors:** Natchaya Kunanitthaworn, Oramai Mueangmo, Jutamad Saheng, Worawan Wongjak, Tanin Lertsiriladakul, Tanachot Chaito, Pasawat Nantarat, Tavitiya Sudjaritruk

**Affiliations:** 1 Division of Infectious Diseases, Department of Pediatrics, Faculty of Medicine, Chiang Mai University, Chiang Mai, Thailand; 2 Clinical and Molecular Epidemiology of Emerging and Re-emerging Infectious Diseases Research Cluster, Faculty of Medicine, Chiang Mai University, Chiang Mai, Thailand

**Keywords:** Epidemiology, Paediatric research

## Abstract

This cross-sectional study aimed to assess seroprevalence of hepatitis A virus (HAV) antibodies and identify factors associated with HAV seropositivity among children and adolescents aged 1–18 years who resided in Chiang Mai, Thailand. Sociodemographic characteristics, sanitation/hygiene, and history of HAV vaccination were collected. Anti-HAV IgG antibody was determined, and a level ≥ 1.0 S/CO defined HAV seropositivity. We enrolled 300 participants; median age 8.7 years, 54% male, and 13% overweight (BMI z-score: + 1 to + 2 standard deviation [SD]). Sixty-five participants (22%) were vaccinated against HAV. Overall, 84/300 participants (28%) demonstrated HAV seropositivity, of whom 55/65 (85%) and 29/235 (12%) were among vaccinated and unvaccinated participants (*P* < 0.001), respectively. Previous HAV vaccination (adjusted odds ratio [aOR] 47.2; 95% CI 20.0–111.8) and overweight (aOR 4.4; 95% CI 1.7–11.3, compared with normal weight [BMI z-score: − 2 to + 1 SD]) were significantly associated with seropositivity of HAV. In the stratified analyses, crowded bedroom (aOR 3.2; 95% CI 1.3–7.8, per one person increase) and overweight (aOR 5.0; 95% CI 1.8–13.7) were factors associated with HAV seropositivity among vaccinated and unvaccinated participants, respectively. Seroprevalence of HAV antibodies in healthy Thai children and adolescents was relatively low. Recommendation of HAV vaccination for these populations, particularly those with high-risk conditions, should be considered.

## Introduction

Hepatitis A virus (HAV) infection is one of the leading causes of viral hepatitis worldwide, with approximately 100 million HAV infections and 1.5 million symptomatic cases each year^1^. HAV is transmitted primarily via the fecal–oral route through consumption of contaminated food and water, or close physical contact with an infectious person (e.g., oral-anal sex)^1,2^. In general, hepatitis A disease is mild and self-limiting which does not cause chronic hepatitis. However, age at natural infection is an important determinant of disease severity and clinical outcomes. Exposure to HAV during childhood is usually associated with asymptomatic infection, whereas HAV infection during adolescence or adulthood mainly results in symptomatic disease, such as fever, loss of appetite, nausea, vomiting, malaise, abdominal pain, jaundice, and pale stools, or may eventually lead to fulminant hepatitis or fatality^2^. Although hepatitis A is a vaccine-preventable disease, an overall case-fatality due to HAV infection ranges from 15,000 to 30,000 deaths per year, with the highest estimates in sub-Saharan African and Asian countries where the vaccination coverage is low^1^.

In the past, hepatitis A was typically endemic in low- and middle-income countries due to large population, low socioeconomic status, poor hygiene and sanitation, and lack of clean water^3,4^. With the growth in household incomes per capita, increased access to safe drinking water, improved hygiene and sanitation systems, rapid urbanization, and HAV vaccine availability in recent decades, several hepatitis A endemic countries have consequently transitioned from high (a country with seroprevalence of HAV infection ≥ 90% by 10 years of age) to intermediate (a country with seroprevalence ≥ 50% by 15 years of age, with < 90% by 10 years of age) or low endemicity levels of disease (a country with seroprevalence ≥ 50% by 30 years of age, with < 50% by 15 years of age) due to a continuous decrease in HAV exposure during childhood^5–12^. This epidemiological change importantly places these countries at increased susceptibility to natural HAV infection among their adult populations whom may suffer greater disease-related morbidity and mortality^8,13^. Therefore, introduction of hepatitis A vaccine to the national immunization program for children and adolescents in such countries is vitally important.

Thailand is a developing country in Southeast Asia which attains upper-middle-income economy. The country has made remarkable progress in poverty reduction, economic growth, as well as hygiene and sanitation standard improvement over the past decades^14^. According to a national seroepidemiological study in 2014 which showed that 50% of Thai people was seropositive for HAV by the age of 42 years, Thailand has transitioned from a low to a very low HAV endemicity (a country with seroprevalence < 50% by 30 years of age)^11^. Yet, there are sporadic cases of hepatitis A among Thai population who might acquire the infection through drinking or eating contaminated food and water, touching contaminated fomites due to poor hygiene and sanitation, or exposure to occasional outbreak events. In 2022, there were 285 confirmed cases of hepatitis A reported to the Bureau of Epidemiology, Division of Disease Control, Ministry of Public Health of Thailand, accounting for an incidence of 0.4 per 100,000 populations^15^. To date, vaccination against HAV in Thai children and adolescents is mostly recommended in the private health sector since the vaccine is currently not part of the Thailand Expanded Programme on Immunization (EPI).

Seroprevalence study is a reliable strategy to evaluate the susceptibility rate of population to an infectious agent of concern which would provide important evidence to help establishing the appropriate national immunization policy and recommendations for the country. However, there are currently limited epidemiological data on HAV seropositivity in children and adolescents, particularly among those living in developing countries including Thailand. This study aimed to assess seroprevalence of HAV antibodies and identify factors associated with HAV seropositivity among children and adolescents residing in Northern Thailand, stratified by hepatitis A vaccination status. In brief, the prevalence of HAV seropositivity following vaccination and its associated factors were evaluated among vaccinated participants, whereas the low HAV seropositivity rate and its relevant factors were examined among unvaccinated individuals independently.

## Methods

### Study design and study participants

A cross-sectional study was conducted at the Faculty of Medicine, Chiang Mai University (CMU)—a tertiary care university hospital in Chiang Mai, Thailand. During November 2021 to September 2022, healthy children and adolescents aged between 1 and 18 years old who resided in urban, semi-urban, and rural districts in Chiang Mai—the largest city in northern Thailand (a total area: 20,170 km^2^; a total population: 1.7 million people, including 303,000 children and adolescents aged 1–18 years) were recruited from daycares, nurseries, pre-kindergartens, kindergartens as well as primary, secondary, and vocational schools by convenience sampling technique. Participants who had primary (e.g., X-linked agammaglobulinemia, selective IgA deficiency, IgG subclass deficiency, common variable immunodeficiency) or secondary immune deficiency diseases (e.g., HIV/AIDS, autoimmune disease, leukemia, lymphoma, diabetes), received immunosuppressive agents (e.g., high-dose corticosteroids [prednisolone ≥ 20 mg/day], tacrolimus, cyclosporin, mycophenolate mofetil, azathioprine, cancer chemotherapy), or had been given blood products within the past 6 months by history taking at the screening visit were excluded. This study was approved by the Research Ethics Committee of the Faculty of Medicine, CMU. Prior to study enrollment, participants and/or caregivers were informed about the study objectives, and written informed consents and assents were obtained from all participants and/or their caregivers, as appropriate. All study procedures were performed in accordance with the ethical standards of the institutional research committee on human experimentation and with the Declaration of Helsinki.

### Sample size calculation

The sample size was calculated based on a previously reported seroprevalence of HAV antibodies of 10% among Thai children and adolescents aged 1–20 years in the 2014 national seroepidemiological study (a reference seroprevalance)^11^; and a presumed seroprevalence of 17% anticipated in this study (an expected seroprevalence) with regard to an increased HAV vaccination coverage among study population in the recent years. Thus, with a 90% confidence and a 5% margin of error, a total of 300 participants were required for this study.

### Clinical data collection

Participants and caregivers were interviewed using a study questionnaire. Information regarding sociodemographic characteristics, including age, sex, primary caregiver, parental education level, monthly household income, and place of living, as well as history of liver diseases or viral hepatitis, history of vaccination against HAV from vaccine booklet review or self-reported information, home environment (e.g., type of housing, number of family member living in the same house, number of people sharing the participant’s bedroom), water supply (e.g., drinking water source), toilet (e.g., type of toilet), and personal hygiene (e.g., usage of towel, food services, usage of serving spoon) were collected. In addition, anthropometric parameters were measured, and body mass index (BMI) was calculated by body weight in kilograms divided by the square of height in meters. Then, BMI z-score was computed based on the 2007 World Health Organization (WHO) growth reference^16^, and was categorized to thin (z-score: <  − 2 standard deviations [SD]), normal (z-score: − 2SD to + 1SD), overweight (z-score: >  + 1SD to + 2SD), and obese (z-score: >  + 2SD), according to the WHO suggested cut-offs^16^.

### Blood collection and serological assay

Phlebotomy was performed by well-trained laboratory technicians. Serum samples were extracted and stored at − 20 °C until laboratory analysis. All sera were analyzed, using an automated chemiluminescent microparticle immunoassay (CMIA), for the detection of immunoglobulin G antibody to HAV (anti-HAV IgG; Alinity i HAVAb IgG, Abbott, Wiesbaden, Germany) on the Alinity i system (Abbott Diagnostics, Chicago, IL, USA) according to manufacturer’s instructions. For each sample, the amount of anti-HAV IgG was calculated based on the ratio of the sample relative light unit (RLU) to the cut-off RLU (S/CO). Seropositivity of HAV was defined as anti-HAV IgG level ≥ 1.0 S/CO. The sensitivity and specificity of the test were ≥ 98% and ≥ 99%, respectively.

### Statistical analysis

All statistical analyses were conducted using Stata statistical software, version 17 (StataCorp LP, College Station, TX, USA). Seroprevalence of HAV antibodies was presented as number (n) and percentage (%). Anti-HAV IgG level was reported as the geometric mean concentration (GMC, S/CO) with 95% confidence interval (95% CI). Notably, in this study, the GMC was calculated among participants with HAV seropositivity, and the participants with undetectable anti-HAV IgG levels (< 1.0 S/CO) were excluded. The comparisons of characteristics between participants previously vaccinated *versus* unvaccinated against HAV were conducted using Chi-squared test or Fisher’s exact test (if < 5 observations) for categorical data, and Wilcoxon rank-sum test for continuous data. Additionally, the comparisons of seroprevalence of HAV antibodies and GMC of anti-HAV IgG between vaccinated *versus* unvaccinated participants, stratified by participants’ age groups (1–5 years, 6–10 years, and 11–18 years), were performed using Chi-squared test and log-linear regression analysis, respectively. Univariable logistic regression analysis was conducted to identify factors associated with HAV seropositivity. Covariates with a *P*-value of < 0.20 as well as the well-known prespecified confounding factors, including age, sex, and BMI, were incorporated in the multivariable analysis. The additional logistic regression analysis which stratified study participants by their hepatitis A vaccination status were also performed to further identify the associated factors of HAV seropositivity in each participant group. The magnitudes of association were expressed as crude odds ratio (crude OR) and adjusted odds ratio (aOR) for the univariable and multivariable analyses, respectively. A two-sided *P*-value of < 0.05 was considered statistically significant.

## Results

### Characteristics of study participants

During the study period, a total of 305 children and adolescents were screened, of which 300 were eligible and enrolled (3 participants were excluded due to the receipt of high-dose corticosteroid, and 2 due to autoimmune diseases). The median age of participants was 8.7 years (interquartile range [IQR] 4.8–13.5 years), 54% were male, and 31% had overweight/obesity (BMI z-score: >  + 1SD). Approximately 92% of participants lived with their biological parents, 67% lived in sub-urban or rural areas, and 6% had monthly household income < 300 USD. Five percent of fathers and mothers of participants were illiterate. None of the participants had a previous history of liver diseases, including viral hepatitis, or contacted with a patient with hepatitis A disease prior to enrollment. According to vaccine booklet review and self-reported information, 65 participants (22%) had received at least one dose of HAV vaccine, of which 72% were an inactivated vaccine, 11% were a live-attenuated vaccine, and 17% were unknown type of vaccine. The median duration from the last dose of HAV vaccine to enrollment was 3.1 years (IQR 1.2–6.4 years).

Regarding home environment, a median number of family members living in the same house was 4 persons (IQR 4–5 persons), and a median number of people sharing the participant’s bedroom was 3 persons (IQR 2–4 persons). Flush toilet accounted for 89% of the residential toilets, and almost all participants (99%) used personal towel. The majority of participants (73%) drank commercial bottled water. During the meal, 91% of participants used their own dining plate, and 45% used serving spoon. The comparisons of participants characteristics, including sociodemographic data, home environment, water supply, toilet, and personal hygiene, between participants previously vaccinated *versus* unvaccinated against HAV are summarized in the Table 1. Overall, vaccinated participants had significantly lower BMI z-score, higher parental education level, and higher monthly household income compared with unvaccinated participants (*P* < 0.05).

**Table 1:** Characteristics of study participants, stratified by hepatitis A vaccination status.

Characteristics*	Vaccinated participants (*n* = 65)	Unvaccinated participants (*n* = 235)	*P*^†^
Sociodemographic characteristics			
Age, year	7.8 (4.1–10.8)	9.2 (5.3–14.1)	0.72
Age group			0.24
1.0–5.9 years	23 (35.4)	71 (30.2)	
6.0–10.9 years	26 (40.0)	80 (34.0)	
11.0–18.0 years	16 (24.6)	84 (35.8)	
Sex			0.55
Male	37 (56.9)	124 (52.8)	
Female	28 (43.1)	111 (47.2)	
BMI z-score, SD	− 0.22 (− 1.09 to 1.35)	0.03 (− 0.84 to 1.42)	< 0.001
BMI category			0.85
Thin	5 (7.8)	12 (5.1)	
Normal	40 (61.5)	151 (64.3)	
Overweight	9 (13.8)	29 (12.3)	
Obese	11 (16.9)	43 (18.3)	
Primary caregiver			0.87
Father/mother/both parents	59 (90.8)	216 (91.9)	
Grandparent	5 (7.7)	18 (7.7)	
Others	1 (1.5)	1 (0.4)	
Paternal education level			< 0.001
Primary/secondary school	3 (4.6)	59 (25.1)	
Vocational/high vocational school	15 (23.1)	43 (18.3)	
Bachelor degree or higher	46 (70.8)	107 (45.6)	
Unknown	0 (0)	13 (5.5)	
Illiterate	1 (1.5)	13 (5.5)	
Maternal education level			< 0.001
Primary/secondary school	2 (3.1)	72 (30.7)	
Vocational/high vocational school	6 (9.2)	28 (11.9)	
Bachelor degree and higher	56 (86.2)	119 (50.6)	
Unknown	0 (0)	3 (1.3)	
Illiterate	1 (1.5)	13 (5.5)	
Place of living			0.94
Urban	21 (32.3)	77 (32.8)	
Semi-urban/ rural	44 (67.7)	158 (67.2)	
Monthly household income, USD			< 0.001
< 300	2 (3.1)	15 (6.4)	
300–1000	9 (13.9)	86 (36.6)	
> 1000–1500	18 (27.7)	64 (27.2)	
> 1500	35 (53.9)	67 (28.5)	
Unknown	1 (1.5)	3 (1.3)	
Home environment, water supply and toilet			
Number of family members living in the same house	4 (4–6)	4 (3–5)	0.14
Number of people sharing participant’s bedroom	3 (2–4)	3 (2–4)	0.06
Drinking water source			0.13
Tapped water	16 (24.6)	34 (14.5)	
Commercial bottled water	48 (73.9)	199 (84.7)	
Well/ground water	1 (1.5)	2 (0.8)	
Type of toilet			0.08
Squat toilet	2 (3.1)	27 (11.5)	
Flush toilet	63 (96.9)	204 (86.8)	
Squat and flushed toilet	0 (0)	4 (1.7)	
Personal hygiene			
Usage of towel			0.62
Personally	64 (98.5)	233 (99.15)	
Common/ shared	1 (1.5)	2 (0.85)	
Food services			0.31
Separated dining plate	59 (90.8)	202 (86.0)	
Common dining plate	6 (9.2)	33 (14.0)	
Usage of serving spoon			0.04
Yes	29 (44.6)	139 (59.2)	
No	36 (55.4)	96 (40.9)	

*BMI* body mass index, *SD* standard deviation, *USD* US dollar.

*Data presented as number (percentage) for categorical data, and as median (interquartile range) for continuous data.

^†^Chi-squared test or Fisher’s exact test (less than 5 observations) was performed for the analysis of categorical data, and Wilcoxon rank-sum test was performed for the analysis of continuous data.

### Seroprevalence of antibodies against hepatitis A virus

**Figure 1:**
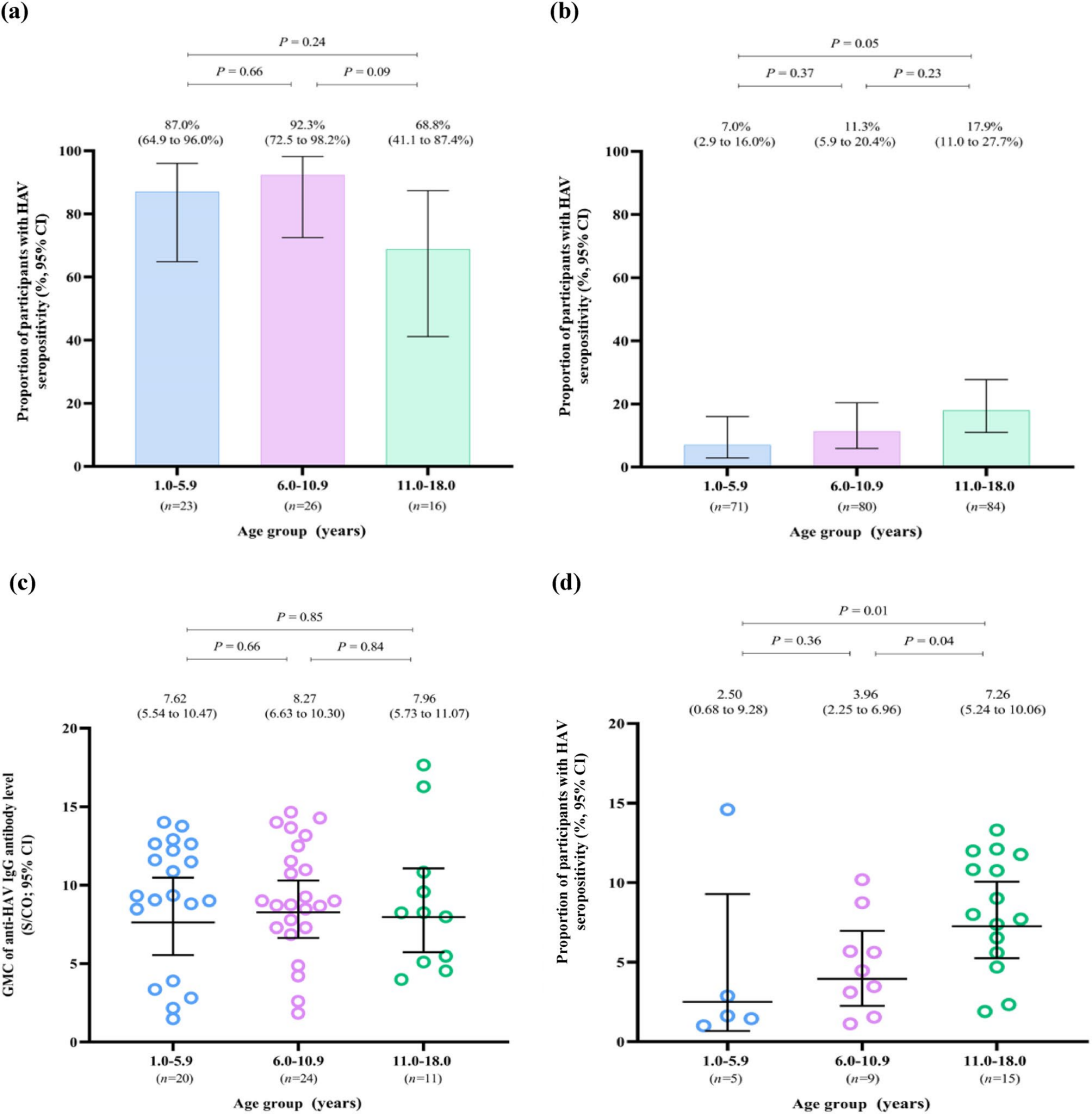
Seroprevalence and geometric mean concentrations of hepatitis A virus antibodies among study participants, stratified by age group and hepatitis A vaccination status of study participants. (**a**) Seroprevalence of hepatitis A virus antibodies among vaccinated participants; (**b**) seroprevalence of hepatitis A virus antibodies among unvaccinated participants; (**c**) geometric mean concentrations of anti-hepatitis A virus IgG among vaccinated participants with hepatitis A virus seropositivity; (**d**) geometric mean concentrations of anti-hepatitis A virus IgG among unvaccinated participants with hepatitis A virus seropositivity. Proportions are shown as colored bar charts, and the corresponding 95% confidence intervals are shown as black horizontal bars. Geometric mean concentrations are shown as colored data points, and the corresponding 95% confidence intervals are shown as black horizontal bars. Chi-squared test was performed to compare the proportions of participants with hepatitis A virus seropositivity between age groups. Log-linear regression analysis was performed to compare the geometric mean concentrations of anti-hepatitis A virus IgG of participants between age groups. *GMC* geometric mean concentration, *HAV* hepatitis A virus, *S/CO* signal to cut-off ratio, *95% CI* 95% confidence interval.

In this study, 84 participants demonstrated seropositivity of HAV, corresponding to the seroprevalence of 28% (95% CI 23–33%), and the GMC of anti-HAV IgG among these participants was 6.78 S/CO (95% CI 5.83–7.89 S/CO). According to participant’s age group, the seroprevalences of anti-HAV IgG antibodies were 27% (GMC: 6.10 S/CO; 95% CI 4.26–8.74 S/CO) among participants aged 1 to 5 years; 31% (GMC: 6.76 S/CO; 95% CI 5.34–8.56 S/CO) in participants aged 6 to 10 years; and 26% (GMC: 7.55 S/CO; 95% CI 6.07–9.39 S/CO) in participants aged 11 to 18 years. Notably, vaccinated participants had significantly higher seroprevalence (85% *versus* 12%; *P* < 0.001) as well as exhibited higher GMC of anti-HAV IgG (7.96 *versus* 5.01 S/CO; *P* = 0.003), compared with those of unvaccinated individuals. The similar findings were also observed across all three participant’s age groups, except for the GMC of anti-HAV IgG among participants aged 11 to 18 years, in which the antibody levels were not different between participant groups (7.96 *versus* 7.26 S/CO; *P* = 0.68). Interestingly, there were no differences in the seroprevalences and the GMCs of HAV antibodies across all three age groups among vaccinated participants as well as unvaccinated individuals, except for the GMC of anti-HAV IgG in unvaccinated participants aged 1 to 5 years (2.50 *versus* 7.26 S/CO; *P* = 0.01) and 6 to 10 years (3.96 versus 7.26 S/CO; *P* = 0.04) *versus* those aged 11 to 18 years (Fig. 1).

### Associated factors of hepatitis A virus seropositivity

In the overall multivariable logistic regression analysis, a history of vaccination against HAV (aOR 47.2; 95% CI 20.0–111.8) and overweight status (aOR 4.4; 95% CI 1.7–11.3, compared with normal BMI) were the significant associated factors of seropositivity of HAV in our participants (Table 2). Furthermore, in the additional analyses stratified by hepatitis A vaccination status of study participants, an increased number of people sharing the participant’s bedroom (aOR 3.2; 95% CI 1.3–7.8, per one person increase) was significantly associated with HAV seropositivity among vaccinated participants, whereas overweight status (aOR 5.0; 95% CI 1.8–13.7, compared with normal BMI) was the significant associated factor among unvaccinated individuals (Table 3).

**Table 2:** Seroprevalence of hepatitis A virus antibodies and associated factors of hepatitis A virus seropositivity among all study participants.

Parameters	Seroprevalence of HAV antibody, n/N (%; 95% CI)	Associated factors of hepatitis A seropositivity					
		Univariable analysis*			Multivariable analysis*		
		Odds ratio	95% CI	*P*	Adjusted odds ratio	95% CI	*P*
Age group							
1.0–5.9 years	25/94 (26.6; 18.6–36.5)	Ref	Ref	Ref	Ref	Ref	Ref
6.0–10.9 years	33/106 (31.1; 23.0–40.7)	1.24	0.67–2.31	0.48	1.64	0.65–4.15	0.29
11.0–18.0 years	26/100 (26.0; 18.3–35.6)	0.97	0.51–1.84	0.93	1.60	0.56–4.66	0.38
Male sex	50/161 (31.1; 24.3–38.7)	1.39	0.83–2.32	0.20	1.70	0.82–3.54	0.16
BMI category							
Normal	47/191 (24.6; 19.0–31.3)	Ref	Ref	Ref	Ref	Ref	Ref
Thin	5/17 (29.4; 11.9–56.3)	1.28	0.43–3.81	0.66	0.70	0.14–3.64	0.67
Overweight	17/38 (44.7; 29.5–61.1)	2.48	1.21–5.09	0.01	4.41	1.71–11.33	0.002
Obese	15/54 (27.8; 17.3–41.4)	1.18	0.60–2.33	0.64	1.15	0.42–3.14	0.78
Previously vaccinated against HAV	55/65 (84.6; 73.5–91.6)	39.07	17.95–85.05	< 0.001	47.23	19.95–111.82	< 0.001
Number of people sharing participant’s bedroom, per one person increase	NA	1.18	0.98–1.42	0.09	1.01	0.74–1.39	0.95
Drinking water source							
Tapped water	20/50 (40.0; 27.2–54.4)	Ref	Ref	Ref	Ref	Ref	Ref
Commercial bottled water	63/247 (25.5; 20.4–31.3)	0.51	0.27–0.97	0.04	0.57	0.22–1.45	0.24
Well/ground water	1/3 (33.3; 0.3–99.0)	0.75	0.06–8.83	0.82	0.53	0.01–32.19	0.76
Not using serving spoon	43/132 (32.6; 25.1–41.1)	1.50	0.90–2.48	0.12	1.21	0.59–2.48	0.61

*BMI* body mass index, *HAV* hepatitis A virus, *NA* not available, *Ref* reference group, *95% CI* 95% confidence interval.

*Univariable logistic regression analysis was performed to identify factors associated with hepatitis A virus seropositivity among all study participants. Covariate with *P* < 0.20, including history of vaccination against HAV, number of people sharing participant’s bedroom, drinking water source, and usage of serving spoon as well as the well-known prespecified confounding factors, including age, sex and body mass index were incorporated in the multivariable logistic regression analysis.

**Table 3:** Associated factors of hepatitis A virus seropositivity, stratified by hepatitis A vaccination status of study participants.

Parameters	Vaccinated participants (*n* = 65)						Unvaccinated participants (*n* = 235)					
	Univariable analysis*			Multivariable analysis*			Univariable analysis*			Multivariable analysis*		
	Odds ratio	95% CI	*P*	Adjusted odds ratio	95% CI	*P*	Odds ratio	95% CI	*P*	Adjusted odds ratio	95% CI	*P*
Age group												
1.0–5.9 years	Ref	Ref	Ref	Ref	Ref	Ref	Ref	Ref	Ref	Ref	Ref	Ref
6.0–10.9 years	1.80	0.27–11.86	0.54	2.52	0.29–22.28	0.41	1.67	0.53–5.25	0.38	1.62	0.48–5.43	0.43
11.0–18.0 years	0.33	0.07–1.65	0.18	0.71	0.09–5.85	0.75	2.87	0.99–8.34	0.05	1.71	0.44–6.56	0.44
Male sex	1.39	0.36–5.37	0.63	0.90	0.16–5.03	0.91	1.54	0.69–3.43	0.29	1.78	0.75–4.22	0.19
BMI category												
Normal	Ref	Ref	Ref	Ref	Ref	Ref	Ref	Ref	Ref	Ref	Ref	Ref
Overweight	2.00	0.22–18.39	0.54	7.13	0.36–142.72	0.20	4.08	1.58–10.55	0.004	5.00	1.82–13.73	0.002
Obese	2.50	0.28–22.49	0.14	5.47	0.30–98.29	0.25	1.19	0.41–3.49	0.75	1.09	0.35–3.42	0.88
Number of people sharing participant’s bedroom, per one person increase	2.47	1.31–4.67	0.005	3.23	1.34–7.80	0.009	0.67	0.48–0.95	0.02	0.70	0.44–1.12	0.14

*BMI* body mass index, *Ref* reference group, *95% CI* 95% confidence interval.

*Univariable logistic regression analysis was performed to identify associated factors of hepatitis A virus seropositivity among study participants. Covariate with *P* < 0.20 which was number of people sharing participant’s bedroom as well as the well-known prespecified confounding factors, including age, sex and body mass index were incorporated in the multivariable logistic regression analysis.

## Discussion

Among children and adolescents living in Northern Thailand, of whom 22% previously received at least one dose of hepatitis A vaccine, the seroprevalence of HAV antibodies was approximately 28%. Focusing on vaccinated participants, the seroprevalence of HAV antibodies was 85%; but reduced to 12% among unvaccinated individuals. Notably, the HAV seroprevalences were comparable across all three age groups in vaccinated as well as unvaccinated individuals. Overall, the important associated factors of HAV seropositivity in our participants included a history of vaccination against HAV and overweight status. Among vaccinated participants, crowded bedroom was the significant associated factors, while it was overweight status for unvaccinated participants. The low seroprevalence of HAV antibodies among participants unvaccinated against HAV demonstrated in this study highlights the importance of the recommendation of HAV vaccine for Thai children and adolescents, particularly those with high-risk conditions. This strategy would help to prevent natural HAV infection among these susceptible individuals, which may cause serious hepatic complications, later in life.

The seroprevalence of HAV antibodies illustrated in this study is comparable to those reported in the previous studies conducted in high-income and upper-middle-income countries^17,18^. In a study carried out in Saudi Arabia, a high-income country, the hepatitis A seroprevalence was approximately 24% among hepatitis B virus (HBV), and hepatitis C virus (HCV)-uninfected children and adolescents aged between 2 and 19 years (the proportion of participants vaccinated against HAV was not reported)^17^. Additionally, a study conducted in Turkiye, an upper-middle-income country, revealed that the seroprevalence of HAV antibodies among healthy children and adolescents aged 1 to 18 years (none were vaccinated against HAV) was about 30%^18^. However, the HAV seroprevalence presented in this study tends to be greater than that reported in the Thai national seroepidemiological study conducted in 2014, in which the seroprevalence was approximately 10% among healthy youth aged between 1 and 20 years^11^. The difference in the HAV seroprevalences among Thai youth over a period might be attributed to the dissimilarities in sociodemographic characteristics, personal hygiene, and hepatitis A vaccination coverage in study population as well as the variations of local epidemiology of HAV infections, hepatitis A diseases and outbreaks across the study areas. According to the statistics from the national passive surveillance system of the Bureau of Epidemiology, Ministry of Public Health of Thailand, the Health Region 1—which includes eight provinces located in the upper northern Thailand—was ranked as the third and the second highest incidence of hepatitis A in Thailand (out of the 12 health regions) in 2021 and 2022, respectively; and Chiang Mai was the province with the highest incidence in the region^19^. These statistics might be related to the middle-to-low socioeconomic status, suboptimal hygiene and sanitation practices, and increased exposure to contaminated food and water of people living in this area. Nevertheless, there were no reports of hepatitis A outbreak in this region during the study period^19^. Since HAV vaccine is currently not part of the EPI in Thailand, the national vaccination coverage estimates among Thai population are unfortunately not available. The coverage of HAV vaccination among children and adolescents residing in Chiang Mai, although increases in the recent years, is expected to be much lower when compares to those living in the central region of Thailand, including Bangkok (the capital city).

The seroprevalences and the GMCs of HAV antibodies in our study participants were significantly greater among individuals previously vaccinated against HAV across all three age groups, except for the GMC of anti-HAV IgG for adolescents aged 10 to 18 years. The higher HAV seroprevalence and HAV antibody levels among vaccinated participants confirms the robust immunogenicity of childhood HAV vaccination, both inactivated and live-attenuated vaccines^20^. Although some previous studies demonstrated the sustained seroprotection up to 10–20 years following a complete HAV vaccination course among healthy children and adults^21–23^, we noted the declining trends in the proportion of participants with HAV seropositivity as well as the GMC of anti-HAV IgG among vaccinated adolescents when compared with the younger age groups in this study. These findings might be attributed to the waning of vaccine-elicited antibody levels over time, particularly among individuals who had incomplete HAV vaccination. In contrast, among unvaccinated adolescents, we observed the increased seroprevalence and antibody levels for HAV when compared with the other age groups, as well as the indifference in the antibody levels when compared with vaccinated individuals in the same age group. We hypothesized that some of the unvaccinated adolescents in this study might have already been exposed to HAV and/or have acquired an asymptomatic natural HAV infection during their adolescence^24,25^.

In this study, we found that a previous history of hepatitis A vaccination was significantly associated with HAV seropositivity among our participants. This finding was similar to a cross-sectional study in India, a lower-middle-income country, which revealed that hepatitis A vaccination, together with parental education levels, source of water supply, and history of jaundice, were the significantly associated factors of HAV seropositivity among their Indian children aged between 1 and 5 years^26^. We also found that overweight status was significantly correlated with seropositivity of HAV in our participants, particularly among unvaccinated individuals. Frequent eating habits might be one possible factor that increased their exposure to natural HAV infections. A previous study conducted in Thailand also reported that truncal obesity was a strong associated factor of a better immune response to single-dose live-attenuated HAV vaccine^27^. To date, the plausible mechanisms of the association between overweight/obesity and strong immune response to HAV infection or vaccination are not clearly understood^28^. The previous evidence showed that overweight and obesity statuses could either increase or decrease total lymphocytes and CD4+ T cells, the key players of adaptive immune system, in peripheral blood populations^29–32^. We further noted that crowded bedroom, as a surrogate of over-crowded family, was significantly linked to HAV seropositivity among vaccinated participants, which was consistent with the finding in a study conducted among Turkish children and adolescents aged 1 to 15 years^18^.

Unlike the other studies conducted in upper-middle-income countries^33–35^, this study did not demonstrate any associations of low socioeconomic status or parental education levels with HAV seropositivity among Thai children and adolescents. Since there were a limited number of participants whose monthly household income less than 300 USD (6%) as well as a small number of illiterate parents (5% of fathers and 5% of mothers) in this study, we might have an insufficient statistical power to illustrate the significant associations between these variables.

The seroprevalence of HAV antibodies among our unvaccinated participants was markedly low in all age groups. The absence of immunity to HAV is a significant concern for the suffering of symptomatic infection, fulminant hepatitis, and fatality among adults infected with HAV^36^. Also, since Chiang Mai province is bordered by Myanmar, where hepatitis A is still endemic^25^, there is a potential for disease outbreaks introduced by migrants crossing the border. HAV vaccine, either inactivated or live-attenuated, is well-tolerated, strongly immunogenic, and highly effective to prevent HAV infection. Introducing HAV vaccine into the national immunization programme is one of the effective ways to protect Thai youth against infection as well as prevent future outbreaks in the high-risk areas. However, given the very low HAV endemicity profile in Thailand^11,25^, the recommendation of HAV vaccine among people at increased risk of infection or its consequences, including people with chronic liver diseases, men who have sex with men, people who use injection or non-injection drugs, patients with clotting-factor disorders, people at risk of occupational exposure, and international travelers to high or intermediate endemicity countries, might alternatively be a cost-effective strategy for the country^36^.

This study contains some limitations. Firstly, the information regarding previous vaccination collected by self-report as well as personal hygiene, and liver disease or viral hepatitis collected by interview might be subjected to recall bias. Also, the details about hand hygiene (e.g., hand washing before meals and after using toilet, and using alcohol gel hand rub) among the study participants and their family members were not collected. In addition, the data on type of HAV vaccine that the participants received was missing in some individuals that limited our ability to specify the proportion of participants receiving complete HAV vaccination and compare HAV seropositivity between participants receiving inactivated *versus* live-attenuated HAV vaccines. Furthermore, using convenience sampling method to recruit study participants, the key epidemiological factors such as age distribution, socioeconomic status, and vaccine coverage rates were not taken into account; and thus, selection bias and non-representative sample might be possible. The lengthy enrollment period due to COVID-19 pandemic also might confound our HAV seroprevalence as a result of seasonal variation of hepatitis A over the period. Lastly, since this study was conducted among children and adolescents residing in Northern Thailand, our findings might not be generalizable to other regions of Thailand as well as other countries where the population and country characteristics are different.

## Conclusions

The seroprevalence of HAV antibodies in healthy children and adolescents living in Northern Thailand was relatively low, particularly among unvaccinated individuals. Vaccination against HAV has significantly contributed to a higher seropositivity rate with a robust antibody response. Therefore, the recommendation of HAV vaccine for Thai children and adolescents, particularly individuals with high-risk conditions, is a crucial strategy to prevent natural HAV infections and serious hepatic complications later in life.

## Data Availability

The datasets generated during and/or analyzed during the current study are not publicly available, but are available from the corresponding author on reasonable request.
